# Eye Movement Study on Attention Bias to Body Height Stimuli in Height Dissatisfied Males

**DOI:** 10.3389/fpsyg.2017.02209

**Published:** 2017-12-22

**Authors:** Fuguo Chen, Jie Liu, Shuanghong Chen, Hong Chen, Xiao Gao

**Affiliations:** ^1^Key Laboratory of Cognition and Personality, Ministry of Education, Southwest University, Chongqing, China; ^2^Faculty of Psychology, Southwest University, Chongqing, China

**Keywords:** height dissatisfaction, eye movement, attention bias, negative physical self, dot-probe task

## Abstract

The present study investigated attention bias in response to height-related words among young men in China. 47 [26 high height dissatisfied (HHD) and 21 low height dissatisfied (LHD)] men performed a dot-probe task. Eye movement (EM) recordings showed that compared to LHD men, HHD men had an avoidance bias in response to height-related words, which was revealed by less frequent first fixations on both tall-related and short-related words, and showed significantly shorter first fixations on short-related words. There was no other significant difference in EM indices (i.e., first fixation latency and gaze duration) between two groups. In addition, HHD participants were significantly slower than LHD participants when responding to probes preceded by short-related words, while there was no difference when probes were preceded by tall-related or neutral words. In sum, the present results indicate that HHD men selectively avoid cues related to short height.

## Introduction

Negative physical self (NPS), also known as body dissatisfaction or body image disturbance, is defined as personal negative cognition, experienced negative emotions, and behavioral regulations (Baker et al., 1995) related to weight, appearance, height, and whole-body dissatisfaction. NPS involves cognitive emotions, behavior, and projection (Chen et al., 2006). One dimension of NPS is negative physical self-shortness (NPS-S), which is specifically related to stature dissatisfaction. NPS-S consists of negative cognition about one’s own height (thinking himself/herself is too short), negative emotions (dissatisfaction with height), and related behavioral regulations (e.g., exercise or medication to increase height). Prior studies have found that body dissatisfaction is a serious problem in western society. Compared with men, Women are more concerned about their figures and appearance, have higher expectations for their appearance and become dissatisfied with their weight and stature more easily (Feingold and Mazzella, 1998; Thompson and Stice, 2001; Phares et al., 2004). For males, greater height is relevant to masculinity, charm and superior attraction (Gillis and Avis, 1980; Feingold, 1982; Hensley and Cooper, 1987; Jackson and Ervin, 1992; Hensley, 1994), which signifies a genetic advantage and power (Judge and Cable, 2004). Therefore, compared with taller men, shorter men may date fewer women (Sheppard and Strathman, 1989; Pawłowski, 2003; Prokop and Fedor, 2011) and receive fewer responses to marriage proposals (Lynn and Shurgot, 1984; Pawłowski and Koziel, 2002).

The influences of short stature on social interaction and personal development also exist in non-western cultures. For example, in China, although illegal, there is height discrimination, some jobs require a minimum height of 170 cm (e.g., bank staff, lawyers and airline staff) (Watts, 2004). A 10-year longitudinal study (2001–2010) in China indicated that shorter workers received lower wages than taller worker for the same job (Gao and Smyth, 2012). And height dissatisfaction in males is a common phenomenon in Asia (Park et al., 2003; Chen et al., 2006; Jackson and Chen, 2008a; Lee et al., 2008; Chen and Jackson, 2009a; Kim and Park, 2009). A large-scale survey on body dissatisfaction suggested that Chinese adolescents were more dissatisfied with stature than with obesity or appearance (Chen et al., 2006).

Prior studies have shown that shorter people or people with height dissatisfaction had lower appearance self-esteem, self-esteem, overall figure satisfaction (Chen et al., 2006; Jackson and Chen, 2008a,b; Tiggemann et al., 2008), general self-esteem (Judge and Cable, 2004; Chen et al., 2006) and subjective happiness (Carrieri and De Paola, 2012). Dieting and exercise can affect weight and body figure, but they have little effect on height, and as a result, height-dissatisfied adults may try expensive and risky leg-lengthening surgery (Watts, 2004; Campens et al., 2010).

Recently, NPS studies have focused on the attention bias of people with weight dissatisfaction, maladjusted body images and eating disorders. Behaviors and EM studies are most frequently cited. People with a maladjusted body image have an attention bias against words and images of body weight, shape-related and food. By using the emotion Stroop color naming task, some studies have shown that compared to healthy participants, participants with bulimia nervosa (BN) and anorexia nervosa (AN) had significantly longer reaction time to food, body shape, and body weight-related words (Fairburn et al., 1991; Cooper and Fairburn, 1992, 1993; Cooper et al., 1992; Davidson and Wright, 2002; Dana et al., 2004) as well as food pictures (Stormark and Torkildsen, 2004). The dot-probe task had also been used to study attention bias in NPS, in which body weight/shape-related or food-related stimuli (words or photographs) were presented on one side of the computer screen while neutral stimuli were presented on the other side. Following stimuli presentation, a probe (e.g., single letter “E” or “F”) was presented immediately in either the location once occupied by body/food or neutral stimulus. Participants were instructed to identify the probe as fast as possible. Compared with healthy participants, people with eating disorders were more likely to pay immediate attention toward negative figure words (e.g., “obesity”) or pictures of obese figures, and avoid positive figures words (e.g., “slim”) (Rieger et al., 1998; Shafran et al., 2007). These findings supported the theory that people with a negative schema selectively attend to negative information and reject positive information (Vitousek and Hollon, 1990; Vitousek and Orimoto, 1993). In addition, visual search tasks demonstrated that compared with healthy counterparts, people with eating disorders show an accelerative detection of body weight/shape-related information and rapid disengagement from food words/photographs (Smeets et al., 2008). Using a spatial cue paradigm, Feng et al. (2010) proposed that attention maintenance was one component of attention bias of NPS obese people. Using EM tracking techniques, Jansen et al. (2005) found that participants with severe eating disorder symptoms paid more attention to their unattractive body parts than attractive body parts. The contrary was that when participants were observing pictures of others, more attention resources were allocated on attractive body parts than unattractive body parts. Interestingly, participants without eating disorder symptoms showed an opposite attention pattern. Using the dot-probe task and EM tracking techniques, Gao et al. (2011, 2012) found that women with weight concerns showed difficulties in disengaging their attention from fat/obese-related stimuli, maintaining attention to obese-related stimuli and stopping avoidance of thin-related words.

The results of studies on obese people with NPS may be influenced by the content of the stimuli. Participants in different subgroups may have diverse processing patterns and pathways when responding to different stimuli. For example, participants with AN and BN disengaged from gazing at NPS schema-related stimuli. However, when the schema was activated, the AN group had a processing bias toward food information while the BN group had a processing bias toward weight and body-shape stimuli (Faunce, 2002). Moreover, using different stimuli might also lead to different processing patterns and pathways even when subgroups are heterogeneous. For example, Gao et al. (2011) found that obese people with NPS showed attention vigilance: attention maintenance pattern toward obese-related words, and attention avoidance pattern toward thin-related words. Some research on obese people with NPS has found that the attention component of the body image disturbance to obese stimuli is attention vigilance, other studies have suggested that the attention component is attention maintenance. Attention vigilance is considered as an inchoate component of attention process while attention maintenance is considered as a terminal attention process. To be specific, weight dissatisfied women were more likely to focus their initial gaze on fatness words, have a shorter mean latency of first fixation on both fatness and thinness words, longer first fixation on fatness words but shorter first fixation on thinness words and shorter total gaze duration on thinness words. Reaction time data showed a maintenance bias toward fatness words in WD women.

If these cognitive biases are the function of distorted body schemata rather than disordered eating, they can also be observed in stature dissatisfied people. Present study used dot-probe task and EM tracking techniques to investigate patterns and processing time of attention bias in response to height-related words in height dissatisfied males. There were few studies on the attention bias of height dissatisfaction, and the current study was based on the results of studies exploring other dimensions (e.g., weight) of body image and hypothesized that HHD males would have the same attention bias pattern as people with weight dissatisfaction or eating disorders (Rieger et al., 1998; Gao et al., 2011, 2012). According to previous findings, the current study hypothesizes that compared with low height-dissatisfied males, high height-dissatisfied males would show attention bias toward height-related words.

## Materials and Methods

### Participants

Undergraduate and graduate male students at Southwest University in China were asked to complete the height subscale of NPS and record their demographics (age, weight, height, left-handed or right-handed, existing neurological disease or psychiatric illness, and telephone numbers). Questionnaires were completed during class time. Appointments were made 2 weeks later with men randomly selected from subgroups in which participants scored higher than 2.0 (*n* = 26) and lower than 1.0 (*n* = 21) on the NPS-S. The mean (±SD) NPS-S score of HHD group was 2.5 ± 0.25 which was significantly higher than LHD group (0.38 ± 0.26) [*F*(1,45) = 869.44, *p* < 0.001, η^2^ = 0.95]. The mean age of participants was 21.34 (*SD* = 1.75, range = 18–25). There was no significant difference in age [*F*(1,45) < 0.01, *p* = 0.980, η^2^ < 0.01] and body mass index (BMI) [*F*(1,45) = 0.06, *p* = 0.810, η^2^ < 0.01] between groups. Males in HHD group were significantly shorter (1.66 m ± 0.03) than males in LHD group (1.79 ± 0.03) [*F*(1,45) = 247.94, *p* < 0.001, η^2^ = 0.85]. All participants were right-handed, had normal or corrected-to-normal vision and reported no past and present neurological disease or psychiatric illness. All experimental procedures were approved by the Southwest University Human Ethics Committee and were conducted in accordance with the guidelines of the Declaration of Helsinki. Informed consent was obtained from all the participants before the beginning of the study.

### Experimental Materials and Procedure

The NPS-S (Chen et al., 2006) was used to assess the level of height dissatisfaction, including cognitive-affective component (e.g., “I pay a lot of attention to my height”), projection (other people’s opinion, e.g., “I feel that I am too short in others’ eyes”) and behavior component (e.g., “If there is a way to make myself taller, I will persevere at it”). There were 11 items and each item was scored on a five-point scale (0 = “not at all true” to 4 = “completely true”). A higher total score indicated a higher level of height dissatisfaction. NPS-S had The a high internal consistency (Cronbach α = 0.88; Chen et al., 2006), acceptable test–retest reliability (a repeated test at 9 months provided a test–retest reliability coefficient of 0.78, Chen and Jackson, 2007) and good structure validity (Chen et al., 2006; Jackson and Chen, 2008a,b,c; Chen and Jackson, 2009a,b). The Cronbach α coefficient was 0.89. Participants who scored higher than 2.0 were included in HHD group and those who scored lower than 1.0 were included in LHD group. Participants who scored between 1 and 2 were excluded. Stimulus materials consisted of 35 “tall-related words” (e.g., tall, lanky, long, giant) and 35 “short-related words” (e.g., short, small, tiny, dwarf). Words were gathered via open questionnaires, internet forums and reference books such as the Modern Chinese Dictionary and Ci Hai. Forty undergraduate males (not included in the current experiment) evaluated valence (1 = “very negative” to 5 = “very positive”), relatedness to height (1 = “not related at all” to 5 = “very closely related”) and degree of familiarity (1 = “not familiar at all” to 5 = “very familiar”) of each word. Those with a score over 3 on three scales were chosen as the experimental words. The mean score of the 24 positive height words was 4.24 ± 0.39 and the mean score of the 24 negative height words was 4.13 ± 0.40. Tall-related words and short-related words were similar in terms of familiarity, valence, length and total number of Chinese character strokes (Appendix A).

With the same probability of appearing on one side of monitor, each target word was presented randomly. Simultaneously, a word unrelated to height but with a similar valance, length and total numbers of the Chinese character strokes was presented on the opposite side of the screen. Twenty-four pairs of neutral furniture words were presented on both sides of the screen as filler and six pairs of neutral furniture words were practice trials. All word-pair and target probes were presented pseudo-randomly in order to rule out sequence effects.

There were three types of word-pairs in total presented randomly, including tall-related word-neutral word (T-N), short-related word-neutral word (S-N), and neutral word- neutral word (N-N). Each type had 24 word-pairs. Words in each pair measured 60 mm × 90 mm and the center of each word was separated by 10 cm with a 29°horizontal and 22°vertical visual field.

Apparatus: Eye link 1000 EM tracking system (SR Research, Mississauga, ON, Canada) was used to collect EM data. The eye-tracker sampling rate was 1,000 Hz with a spatial accuracy of 0.1°. Stimuli were presented on a 21-inch, 85-Hz, 1024 pixel × 768 pixel CRT monitor which was connected to a Pentium IV 3.2-GHz host computer. Participants were seated 70 cm away from the monitor screen.

Dot-probe task: At the start of each trial, a white central fixation cross “+” was shown for 1,000 ms on a black screen and then replaced by a word-pair that existed for 1,500 ms. After the offset of each word-pair, a probe was presented where one of the words had appeared. The classical task used one probe on the left or right side of the screen. Participants were required to indicate where the probe appeared by pressing the keyboard. Probes disappeared as soon as participants pressed a key (or after 5,000 ms if no response was made). The time interval between each trial was between 750 and 1,250 ms.

Each participant was required to finish 12 practice trials to become familiar with the experimental procedure. There were 144 trials in total (excluding practice trials), which were separated into two blocks. Each block consisted of 24 trials of each word-pair type (T-N, S-N, and N-N). N-N word-pairs acted as fillers used to mask the experimental intent. In addition, fillers could reduce tediousness such that a middle level cognitive loading existed (Castellanos et al., 2009). Each word-pair was presented twice during the dot-probe task. Probes appeared equally in each side.

### Measures

Behavior: Attention bias scores were calculated via reaction time (RT). The formula adopted was [(H l D r - H r D r) + (H r D l - H l D l)]/2 (MacLeod and Mathews, 1988) where H = height words, D = dot probe, l = left, and r = right. A positive attention bias score indicated an attention orientation toward height words [i.e., shorter RT to probes after height words (effective cues) than probes after neutral words (ineffective cues)]. An attention bias score of zero indicated no attention bias, and a negative score was indicative of avoidance of height words [i.e., longer RT to probes after height words (effective cues) than after neutral words (ineffective cues)].

Preparation and analyses of EM data: saccades that remained stable within a 1° visual angle for at least 100 ms were classified as fixations to that position (Applied Science Group, 2000). Fixations on stimuli were identified as effective when the following conditions were satisfied: during “+” presentation prior, participants fixated at the central region; after the presentation of the stimuli/word-pairs, saccades occurred for at least 100 ms. Fixation latencies shorter than 100 ms were unrelated to the stimuli, and saccades may be spontaneous (Fischer and Weber, 1993); during the presentation of the stimuli/word-pairs, participants fixated at least on one of the words instead of other locations around the screen. Due to some unqualified ineffective fixations, 16.24% trials were excluded from the final analysis.

Based on previous studies (Garner et al., 2006; Castellanos et al., 2009; Gao et al., 2011, 2012), the following four EM indices were recorded in detail below: (1) direction of initial EM after word-pair onset, (2) first fixation latency, (3) first fixation duration, and (4) overall gaze duration. N-N word pairs worked as fillers and hence did not include in data analyses.

Direction bias of initial EM was calculated as total fixation time on a height related word ÷ total time of all trials under height word condition (Garner et al., 2006; Castellanos et al., 2009; Gao et al., 2011, 2012). This score measures the initial attention orientation bias toward a particular stimulus. A score over 50% indicates attention orientation toward height-related words; a score equal to 50% indicates no orientation bias; and a score less than 50% indicates an avoidance of height-related words.

First fixation latency bias was calculated as initial fixation latencies of height-related words – initial fixation latencies of neutral words. This score measures the detection speed of each type of stimulus. A positive score indicates slower detection of height-related words; a score of zero indicates no attention bias and a negative score indicates a detection bias toward height-related words.

First fixation duration bias was calculated by first fixation duration to height-related words – first fixation duration to neutral words. This score measures the initial maintenance of attention toward height-related stimuli. A positive scores reflects initial attention maintenance of height-related words; a score of zero indicates no attention bias and a negative score indicates an initial attention avoidance of height-related words.

Overall gaze duration bias was calculated by overall gaze duration on height-related words ÷ overall duration of the trial (Castellanos et al., 2009). This score measures the overall maintenance of attention. A score over 50% reflects overall attention maintenance of height-related words throughout the entire cognitive process; a score of 50% indicates no overall attention bias, and a score less than 50% indicates an overall attention avoidance of height-related words.

## Results

### Preliminary Analyses

We excluded 16.24% trials which contained false responses. Trails with an RT less than 200 ms and over 2,000 ms, or with ineffective fixations were removed from date analysis. A 2 (groups: HHD vs. LHD) × 3 (word types: tall-related words vs. short-related words vs. neutral words) ANOVA was used and we found no main effect of word-type, *F*(2,45) = 0.90, *p* = 0.406, η^2^ = 0.02, no main effect of group, *F*(2,45) = 1.76, *p* = 0.191, η^2^ = 0.04, and no word-type × group interaction, *F*(2,45) = 0.71, *p* = 0.480, η^2^ = 0.02 on error ratio.

### Attention Bias in Response to Height Stimuli in HHD and LHD Subgroups

**FIGURE 1:**
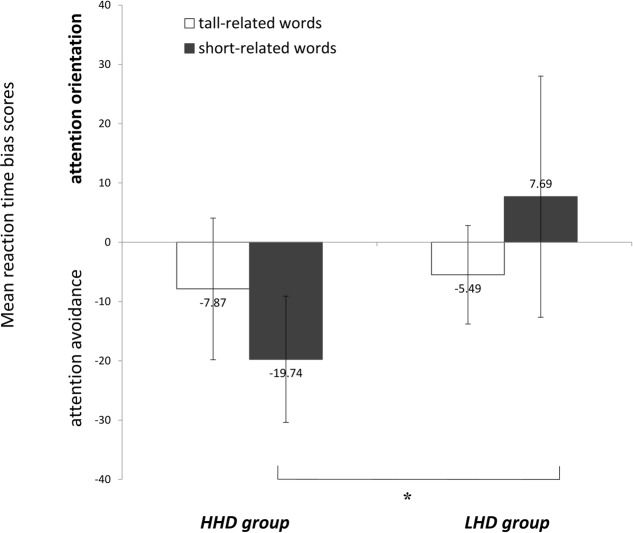
Reaction time of tall- and short-related words among HHD group and LHD group. ^∗^*p* < 0.05.

A 2 (groups: HHD vs. LHD) × 2 (word-types: tall-related words vs. short-related words ANOVA was run on RT, based on MacLeod and Mathews (1988), to investigate attention bias (**Table 1**). All *p*-values were adjusted by the Greenhouse-Geisser method for between group comparisons (Greenhouse and Geisser, 1959). There was no main effect of word-type, *F*(1,45) = 0.01, *p* = 0.929, η^2^ < 0.01, but a significant main effect of group, *F*(1,45) = 7.43, *p* = 0.009, η^2^ = 0.14. There was a marginally significant group × word-type interaction, *F*(1,45) = 3.00, *p* = 0.090, η^2^ = 0.06. Simple effects analyses indicated that HHD group had longer RT to short-related words compared to the LHD group (negative response bias values for the HHD group, and positive values for the LHD group), *F*(1,45) = 6.85, *p* = 0.012, η^2^ = 0.13, suggesting that HHD males, compared to LHD males, have a stronger attention avoidance to short-related words. There was no significant difference between groups when probes were presented in the same location as tall -related words, *F*(1,45) = 0.10, *p* = 0.749, η^2^ < 0.01. An independent sample *t*-test was applied to attention bias scores of each word-type in each subgroup (HHD and LHD) separately. The HHD group showed an attention avoidance from short-related words, *t*(25) = 3.82, *p* = 0.001, *d* = 0.75, but no attention avoidance or orientation bias toward tall-related words, *t*(25) = 1.36, *p* = 0.187, *d* = 0.27, whereas LHD group showed no attention avoidance from short-related words, *t*(20) = 0.79, *p* = 0.440, *d* = 0.18, no orientation bias toward short-related words, *t*(20) = 1.38, *p* = 0.183, *d* = 0.31 (**Figure 1**).

**Table 1:** Reaction time (ms) of height-related words in the HHD and LHD subgroups.

Word-pair type	Category	HHD (*n* = 26)		LHD (*n* = 21)	
		*M*	*SD*	*M*	*SD*
Short–neutral	Short	652.48	116.47	631.53	97.79
	Neutral	632.73	120.58	639.22	98.62
Tall–neutral	Tall	640.80	118.45	625.88	99.79
	Neutral	632.93	122.23	620.39	98.30

### EM Results

A 2 (groups: HHD group vs. LHD group) × 2 (word types: tall-related words vs. short-related words) ANOVA was run on each of the four EM indices.

#### Direction Biases of Initial EM after Word-Pair Onset

**FIGURE 2:**
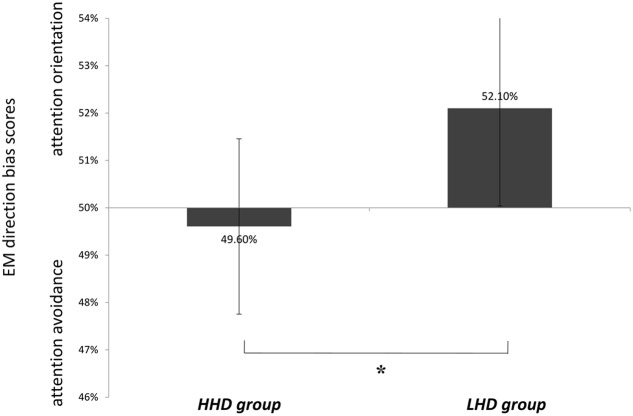
Eye-movement direction bias scores among HHD group and LHD group. ^∗^*p* < 0.05.

There was a marginally significant main effect of group on initial EM after word-pair onset, *F*(1,45) = 3.29, *p* = 0.076, η^2^ = 0.07. The effect size, however, was moderate and acceptable (**Figure 2**). An LSD *t*-test showed that the direction bias of initial EM of HHD group was less than 50% while LHD group was greater than 50%, suggesting that HHD group had an initial avoidance bias from height-related words. There was a significant main effect of word type, *F*(1,45) = 0.45, *p* = 0.504, η^2^ = 0.01,and a group × word-type interaction, *F*(1,45) = 0.16, *p* = 0.696, η^2^< 0.01.

#### First Fixation Latency Biases

There was no significant main effect of word-type, *F*(1,45) = 0.06, *p* = 0.809, η^2^ < 0.01, group *F*(1,45) = 0.20, *p* = 0.654, η^2^ < 0.01, or group × word-type interaction, *F*(1,45) = 0.75, *p* = 0.391, η^2^ = 0.02 on first fixation latency.

#### First Fixation Duration

**FIGURE 3:**
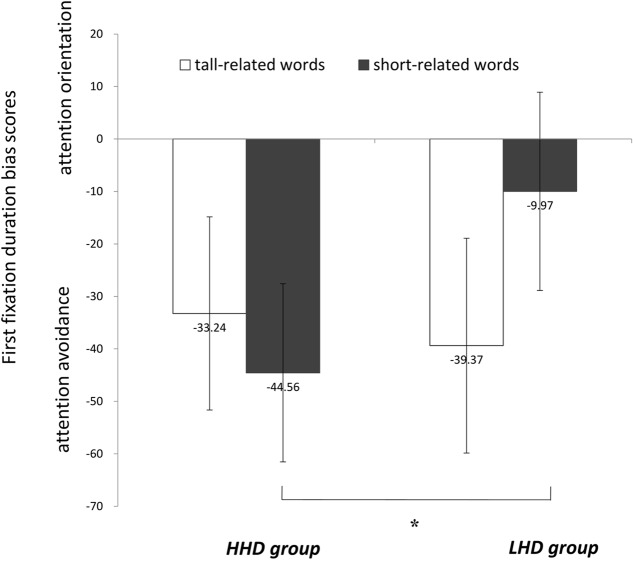
First fixation duration bias scores among HHD group and LHD group. ^∗^*p* < 0.05.

There was a significant group × word-type interaction on first fixation duration, *F*(1,45) = 6.31, *p* = 0.012, η^2^ = 0.12. A simple effect analysis showed that HHD group had a shorter first fixation duration toward short-related words than LHD group, *F*(1,45) = 7.53, *p* = 0.009, η^2^ = 0.14 (**Figure 3**). An independent sample *t*-test was applied to first fixation duration bias scores of each word-type in HHD and LHD groups separately. The results showed that participants in HHD group showed attention avoidance of short-related words, *t*(25) = 5.19, *p* < 0.001, *d* = 1.02, and tall-related words, *t*(25) = 3.74, *p* = 0.001, *d* = 0.73. In other words, HHD group was more likely to disengage gaze from height words than neutral words. LDH group showed significant attention avoidance of tall-related words, *t*(20) = 3.74, *p* = 0.001, *d* = 0.73, but no attention biases against short-related words, *t*(20) = 1.09, *p* = 0.288, *d* = 0.24. There were no main effect of word-type, *F*(1,45) = 1.25, *p* = 0.271, η^2^ = 0.03 or group, *F*(1,45) = 1.89, *p* = 0.176, η^2^ = 0.04, on first fixation duration.

#### Overall Gaze Duration

There were no significant main effect of word-type, *F*(1,45) = 0.32, *p* = 0.577, η^2^ < 0.01, group, *F*(1,45) = 1.89, *p* = 0.176, η^2^ = 0.04, or word-type × group interaction, *F*(1,45) = 0.16, *p* = 0.693, η^2^ < 0.01, on overall gaze duration (**Tables 2**, **3**).

**Table 2:** Eye movement data of height-related words in the HHD and LHD groups.

EM Index and word-pair type	Category	HHD group		LHD group	
		*M*	*SD*	*M*	*SD*
**First fixation direction bias (%)**					
Short—neutral pairs	Short	49.45	4.89	51.51	7.06
Tall—neutral pairs	Tall	49.76	1.2	52.70	1.34
**First fixation latency (ms)**					
Short—neutral pairs	Short	348	54	338	46
	Neutral	345	58	330	32
	Bias scores	3	26	8	29
Tall—neutral pairs	Tall	349	49	323	32
	Neutral	344	48	320	33
	Bias scores	5	19	3	12
**First fixation duration (ms)**					
Short—neutral pairs	Short	258	67	285	70
	Neutral	291	75	295	88
	Bias scores	-33	45	-9	41
Tall—neutral pairs	Tall	246	43	263	68
	Neutral	291	64	302	95
	Bias scores	-45	43	-39	48
**Overall gaze duration bias (%)**					
Short—neutral pairs	Short	48.81	3.55	48.8	4.01
Tall—neutral pairs	Tall	48.74	2.19	48.4	4.01

**Table 3:** Reaction bias scores on height-related words in experiment group and control group.

	*N*	Mean	Standard deviation	Standard error mean
**Experimential group**				
T-orienting	26.00	-7.87	29.60	5.81
Sorienting	26.00	-19.74	26.35	5.17
**control group**				
T-orienting	21.00	-5.49	18.24	3.98
Sorienting	21.00	7.69	44.69	9.75

## Discussion

In the present study, HHD group presented a predominant attention avoidance of negative height stimuli (e.g., short-related words) compared with LHD group, supporting the cognitive behavioral model of body image disturbance (Williamson, 1996; Williamson et al., 2000). Specifically, males with HHD had inchoate avoidance of height-related words illustrated as the initial fixation direction away from tall and short -related words, first fixation attention avoidance of short-related words and RT showing a late attention avoidance of short-related words. These results were opposite to earlier studies on weight dissatisfaction and eating disorders which showed attention vigilance and maintenance toward weight-related words (Williamson, 1996; Williamson et al., 2000). One possible explanation is that we only enrolled male participants in the current study and their height-related schemata were mainly situation-induced. Therefore, they did not show attention bias toward height-related words.

Shafran et al. (2007) reported no significant differences between high weight dissatisfaction group and low weight dissatisfaction group in response to thin-related stimuli. The results of the current study were in agreement with that finding. Shafran et al. (2007) theorized that the lack of group differences may be due to the lack of connection between thin stimulus and high weight dissatisfaction. Others (Koster et al., 2005) have theorized that attention avoidance was only induced by highly threatening stimuli. In the present study, HHD participants hold a short NPS schema through negative cognition, related projection, emotions, and behaviors. Consequently, compared with tall-related words, short-related words were perceived more threatening. At the early stage of stimulus presentation, NPS-S schema of males with HHD may be automatically activated. When stimulus duration was 1,500 ms, it may invoke voluntary strategic avoidance in behavioral trials. Additionally, compared with LHD group, participants in HHD group showed an initial fixation avoidance of tall-related words (less frequent first fixations on tall-related words). These results suggested that automatic activation of NPS-S schema may result in avoidance of height-related information (i.e., tall-related words and short related words). In this case, all height-related words (both tall-related and short-related words) were perceived as threatening and could cause discomfort. These results supported the cognitive-behavior model of body image (Williamson, 1996; Williamson et al., 2000).

The results of the present study suggested that HHD males had both early (as seen through the EM indices) and late (as seen through the behavioral index) stage attention avoidance of short-related words. However, the underlying mechanism of avoidance from short-related words was still unclear. Contrary to these findings, prior research had found that both clinical and non-clinical female participants showed attention orientation toward obese stimuli that were perceived as threatening (Shafran et al., 2007; Smeets et al., 2008; Glauert et al., 2010; Gao et al., 2011, 2012). A large survey (*N* = 1990) on the coping strategies adopted by adolescents to deal with stress found that males were more likely to choose avoidant strategies to distract attention from negative emotions and problems (Eschenbeck et al., 2007) while females preferred to adopt the coping strategies to direct face their inner negative affections and problem-related thoughts. This kind of strategies could in turn augment negative affections while avoidant strategies could reduce negative affections (Butler and Nolen-Hoeksema, 1994), which may explain why females were more likely to become depressed than males after suffering from negative events (Nolen-Hoeksema, 1987; Cyranowski et al., 2000). This reasoning may also explain why females with obese NPS had a pattern of attention orientation rather than attention avoidance of weight/figure-related stimuli. Therefore, the attention patterns observed in the current study may reflect males’ strategic avoidance of threatening stimuli. Further discussion is necessary.

Mogg et al. (2004) found that the exposure to threatening or disgusting stimuli for long durations could cause attention avoidance. Moreover, Cisler and Koster (2010) argued that attention avoidance at later stages of processing resulted from emotional management and goal adjustment, reflecting a strategic attempt to deal with negative emotions. In the current study, the participants in HHD group who scored high on the NPS-S also possessed a high degree of negative cognition and negative behavioral regulation in relation to height.

The process of perceiving height-related words (especially short-related words) as threatening or disgusting was involuntary. Exposure to threatening or disgusting stimuli for 1,500 ms would be adequate to produce a tendency to escape or avoid (Bradley et al., 1999). Future studies on how males with HHD appraise short-related words are warranted.

In addition to providing evidence that the cognitive-behavioral model of body image disturbance is applicable to males with HHD, the results of the current study are also important in more general terms. Similar to attention avoidance in anxious people (Thorpe and Salkovskis, 1999), the avoidance of height-related stimuli may result in short-term self-esteem protection and anxiety decrease in males with HHD. However, ultimately, the avoidance of threatening information did not provide a long-term resolution and conversely may even cause enduring symptoms (Koster et al., 2005). Cognitive therapy aimed at developing alternative opinions about height (e.g., “Though I am shorter than other people, this is not important.”) may provide a long-term solution to NPS-S.

There is a minimum height requirement for some high-income positions (i.e., bank staff, lawyers, and airline company staff) and shorter workers are paid less than taller counterparts in China (Gao and Smyth, 2012). The current study included a cultural component to some extent and studies on avoidance of height-related words in Western cultures are warranted.

## Author Contributions

FC and JL designed the study and wrote the protocol. HC conducted literature searches and provided summaries of previous research studies. XG was responsible for data statistics. SC revised the language and all authors have approved the final manuscript.

## Conflict of Interest Statement

The authors declare that the research was conducted in the absence of any commercial or financial relationships that could be construed as a potential conflict of interest.

## Abbreviations

EM: eye movement
HHD: high height dissatisfied
LHD: low height dissatisfied
NPS-S: Negative Physical Self Scale – Stature Concerns subscale
