# Supplementary material for: Eye Movement Study on Attention Bias to Body Height Stimuli in Height Dissatisfied Males
Source: Front Psychol. 2017 Dec 22;8:2209. doi: 10.3389/fpsyg.2017.02209 (PMC5744235; doi:10.3389/fpsyg.2017.02209)
Supplement: Supplementary file 1 [file Data_Sheet_1.pdf]

(**Appendix A**).

With the same probability of appearing on one side of monitor, each target word was presented randomly. Simultaneously, a word unrelated to height but with a similar valance, length and total numbers of the Chinese character strokes was presented on the opposite side of the screen. Twenty-four pairs of neutral furniture words were presented on both sides of the screen as filler and six pairs of neutral furniture words were practice trials. All word-pair and target probes were presented pseudo-randomly in order to rule out sequence effects.

There were three types of word-pairs in total presented randomly, including tall-related word-neutral word (T-N), short-related word-neutral word (S-N), and neutral word- neutral word (N-N). Each type had 24 word-pairs. Words in each pair measured 60 mm × 90 mm and the center of each word was separated by 10 cm with a 29°horizontal and 22°vertical visual field.

Apparatus: Eye link 1000 eye movement tracking system (SR Research, Mississauga, Ontario, Canada) was used to collect EM data. The eye-tracker sampling rate was 1,000 Hz with a spatial accuracy of 0.1°. Stimuli were presented on a 21-inch, 85-Hz, 1024 × 768 pixel CRT monitor which was connected to a Pentium IV 3.2-GHz host computer. Participants were seated 70 cm away from the monitor screen.

Dot-probe task: At the start of each trial, a white central fixation cross "+" was shown for 1,000 ms on a black screen and then replaced by a word-pair that existed for 1,500 ms. After the offset of each word-pair, a probe was presented where one of the words had appeared. The classical task used one probe on the left or right side of the screen. Participants were required to indicate where the probe appeared by pressing the keyboard. Probes disappeared as soon as participants pressed a key (or after 5,000 ms if no response was made). The time interval between each trial was between 750 ms to 1,250 ms.

Each participant was required to finish 12 practice trials to become familiar with the experimental procedure. There were 144 trials in total (excluding practice trials), which were separated into two blocks. Each block consisted of 24 trials of each word-pair type (T-N, S-N, and N-N)). N-N word-pairs acted as fillers used to mask the experimental intent. In addition, fillers could reduce tediousness such that a middle level cognitive loading existed (Castellanos et al., 2009). Each word-pair was presented twice during the dot-probe task.

Probes appeared equally in each side.

Appendix A. word list: english and Chinese

group	word		mean	group	word		mean
	Chinese	english			Chinese	english	
1	伟岸	stalwart	3.26	2	短小	short	3.21
1	长腿	dolichocnemia	3.73	2	潘长江	Pan Changjiang who is a short actor	3.35
1	挺拔	Tal land straight	3.73	2	五短身材	Be short of stature	3.58
1	易建联	Yi Jianlian who is a basketball player	3.80	2	短腿	short legs	3.60
1	魁梧	tall and strong	3.78	2	武大郎	Wu Dalang who is a short man	3.70
1	彪形大汉	Burly chap	3.95	2	五尺男儿	Five feet man	3.75
1	魁梧轩昂	Tall and imposing	3.95	2	矮挫	short and ugly	3.78
1	魁伟	burly	3.98	2	小个	A small	4.13
1	七尺男儿	Seven feet man	4.18	2	个子短小	Short and short	4.20
1	大个子	Big man	4.23	2	矮小个子	Short stature	4.28
1	姚明	Yao ming	4.40	2	低矮	Low	4.30
1	巨人	Giant	4.30	2	小个子	Little chap	4.33
1	高壮	Tall and Strong	4.33	2	短小身材	Short stature	4.33
1	高大魁梧	Tall and big	4.48	2	身躯矮小	Short stature	4.35
1	人高马大	big and tall	4.55	2	身材矮小	Short stature	4.38
1	高大	Tall and big	4.55	2	侏儒	Dwarf	4.41
1	高个子	Tall man	4.55	2	小矮个	Shorty	4.40
1	高大威武	Mighty tall	4.50	2	个子低矮	Low stature	4.43
1	大高个	Tall and big	4.60	2	矮子	Dwarf	4.45
1	长得高	Grow tall	4.60	2	长得矮	Grow short	4.45
1	身材高大	Tall and tall	4.63	2	矮个儿	Short	4.45
1	个子高大	Tall	4.60	2	矮个子	Short	4.45
1	高个	Tall	4.60	2	矮个	Short	4.45
1	高个儿	Tall man	4.63	2	矮小	short and small	4.46
